# The dual role of N6‐methyladenosine modification of RNAs is involved in human cancers

**DOI:** 10.1111/jcmm.13804

**Published:** 2018-07-24

**Authors:** Liujia He, Jiangfeng Li, Xiao Wang, Yufan Ying, Haiyun Xie, Huaqing Yan, Xiangyi Zheng, Liping Xie

**Affiliations:** ^1^ Department of Urology First Affiliated Hospital School of Medicine Zhejiang University Hangzhou China

**Keywords:** ALKBH5, cancer, epigenetics, FTO, METTL3, N6‐methyladenosine (m^6^A)

## Abstract

As the most abundant and reversible RNA modification in eukaryotic cells, m^6^A triggers a new layer of epi‐transcription. M^6^A modification occurs through a methylation process modified by “writers” complexes, reversed by “erasers”, and exerts its role depending on various “readers”. Emerging evidence shows that there is a strong association between m^6^A and human diseases, especially cancers. Herein, we review bi‐aspects of m^6^A in regulating cancers mediated by the m^6^A‐associated proteins, which exert vital and specific roles in the development of various cancers. Generally, the m^6^A modification performs promotion or inhibition functions (dual role) in tumorigenesis and progression of various cancers, which suggests a new concept in cancer regulations. In addition, m^6^A‐targeted therapies including competitive antagonists of m^6^A‐associated proteins may provide a new tumour intervention in the future.

## 1 INTRODUCTION

Epigenetics, a branch of genetics with stable heritable traits, is defined as the functionally relevant changes or gene expression alteration based on DNA methylation, histones modification, chromatin remodelling, gene silencing, RNA modification, *etc*.^1^ There are several identified epigenetic modifications, paving the way for cell growth, differentiation, self‐renewal and division. A common epigenetic mark is 5‐methylcytosine,^2^ which has been termed the “fifth base” with potential functions on the control and regulation of gene transcription and protein translation by recruiting DNA‐binding proteins.^2^, ^3^ Similarly, a multitude of modifications, termed as epi‐transcriptomics, are identified on RNA from all three kingdoms of life.^4^ They are N6‐methyladenosine (m^6^A), N7‐methylguanosine, 5‐methylcytosine and N1‐methyladenosine, *etc*. Recent studies have illustrated that m^6^A modification is a highly abundant and conservative RNA modification in eukaryotic cells.^5^, ^6^ In addition, changes in m^6^A modification are observed to be involved in multiple cellular processes, which may have impacts on several human diseases.^7^, ^8^ Recently developed methods have enabled researchers to determine the precise location and abundance of m^6^A residues and their implication in human diseases, especially in cancers.^7^, ^9^ Herein, we provide an updated review regarding the critical regulatory effects of m^6^A modification in several human cancers, and to improve the understanding of mechanisms of tumour carcinogenesis.

## 2 THE DISCOVERY OF m^6^A AND ITS FUNCTION

### 2.1 The discovery of m^6^A

Despite the early discovery of m^6^A in 1974, the function and mechanism of m^6^A were unclear until fat mass and obesity‐associated protein (FTO) was detected to be a demethylase of m^6^A in 2011.^10^ Since then, increased attention was paid to the fundamental mechanism and biological function of m^6^A significantly.^10^, ^11^ Alpha‐ketoglutarate‐dependent dioxygenase homolog 5 (ALKBH5), the second RNA demethylase, was first reported in 2013.^12^ Both FTO and ALKBH5 belong to the alpha‐ketoglutarate‐dependent dioxygenase family and catalyse m^6^A demethylation in a Fe(II)‐ and alpha‐ketoglutarate‐dependent manner.^13^, ^14^ Analogous to ALKBH5, alpha‐ketoglutarate‐dependent dioxygenase homolog 3 (ALKBH3) has been demonstrated demethylase activity for 1‐methyladenine and 3‐methylcytosine.^15^ Recently, Ueda et al. have reported that m^6^A was also a substrate of ALKBH3.^16^ Interestingly, ALKBH3 shows a special substrate preference for RNA, targeting only m^6^A in tRNA, rather than those in mRNA or rRNA. An early study found two components of m^6^A methyltransferase in HeLa cells, termed as MT‐A and MT‐B.^17^ Subsequently in 1997, a subunit of MT‐A was identified and termed as methyltransferase‐like protein 3 (METTL3).^18^ However, it was until in 2013 that methyltransferase‐like protein 14 (METTL14), the second component of methyltransferase, was identified. Besides, METTL3 and METTL14 belong to two separate families, but are highly homogenous.^19^ Shortly following this identification, Wilms’ tumour 1‐associating protein (WTAP) was identified with the function of supporting the heterodimer core complex of METTL3‐METTL14 to localize into nuclear speckles.^20^ An additional observation in 2014 interestingly demonstrated that KIAA1429 (also known as VIRMA) was substantially required for complete methylation.^21^ After that, researchers found the depletion of VIRMA led to the largest reduction in mRNA methylation among the known writers.^22^ And further studies indicate that VIRMA is engaged in recruiting METTL3‐METTL14‐WTAP at specific site and HAKAI is also an important component of methyltransferase. Besides, ZC3H13 plays a role in anchoring the m^6^A regulatory complex in the nucleus.^23^ Additionally, Jaffrey et al. demonstrated two previously unrecognized components of the m^6^A methylation complex, namely RNA‐binding motif protein 15 (RBM15) and its paralogue RBM15B, in 2016.^24^ And METTL16 is also regarded as the methyltransferase that modifies U6 snRNAs and various non‐coding RNAs.^25^, ^26^ To date, the supposed core component of m^6^A methyltransferase is METTL3‐METTL14‐WTAP‐VIRMA‐HAKAI‐ZC3H13.

The YTH domain family, with its RNA‐binding domains, was the first identified “reader”. Wang et al. indicated that YTH domain family protein 2 (YTHDF2) selectively bound to m^6^A‐containing mRNA, subsequently reducing the stability of the target transcripts and affecting the degradation of the mRNA.^4^ In contrast, YTH domain family protein 1 (YTHDF1) exerts its role in promoting translation efficiency.^27^ In the meantime, the role of YTH domain‐containing protein 1 (YTHDC1) in regulating mRNA splicing has been revealed.^28^, ^29^ In 2017, Shi et al. showed that YTH domain family protein 3 (YTHDF3) promoted translation in synergy with YTHDF1 and affected methylated mRNA decay mediated through YTHDF2.^30^ And recently, researchers have found that YTHDC2 promotes translation efficiency and decreases the mRNA abundance.^31^ Apart from the YTH domain family, certain proteins in the heterogeneous nuclear ribonucleoprotein family also act as “readers”. In 2015, heterogeneous nuclear ribonucleoprotein A2‐B1 (HNRNPA2B1) was first determined to bind to m^6^A‐containing miRNA transcripts and promote primary miRNA processing.^32^ Additionally, it was also found that the binding process between heterogeneous nuclear ribonucleoprotein C (HNRNPC) and substrate RNA was partially mediated by m^6^A modification.^33^

**Figure 1:**
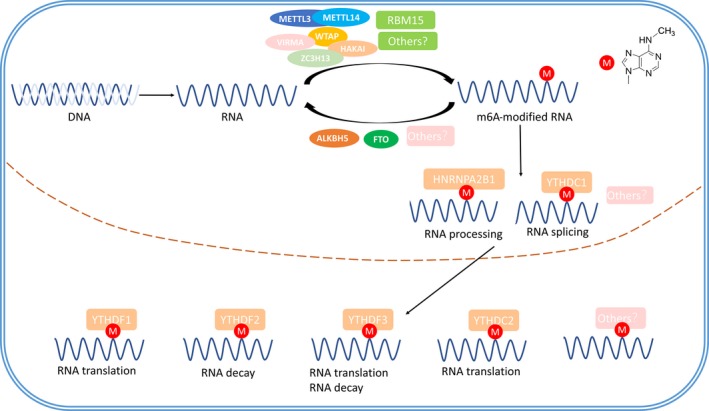
M^6^A modification‐associated proteins and the modification pathways. The methyltransferases (METTL3, METTL14, WTAP, VIRMA, HAKAI, ZC3H13 and RBM15) catalyse RNAs to produce methylation at the N6 position of adenosine, and the demethylases (FTO and ALKBH5) conversely remove methyl groups. Nuclear readers YTHDC1 and HNRNPA2B1 regulate the RNA processing, and cytoplasmic readers YTHDC2, YTHDF1‐YTHDF3 exert roles in m^6^A‐containing mRNA translation and decay.

Following investigations of the m^6^A‐associated proteins complex, we conclude that there is a dynamic reversible process which is composed of the methyltransferase complex, independent demethylases and function executives. The methyltransferases, also known as “writers”, catalyse RNAs to promote and produce methylation at the N6 position of adenosine, and consist of METTL3, METTL14, WTAP, RBM15, VIRMA, HAKAI and ZC3H13. The demethylases, called “erasers”, conversely remove methyl groups to reverse the m^6^A modification and are composed of FTO and ALKBH5. The final function executions are mediated by variable “readers” to determine different downstream effects by recognizing m^6^A sites. Readers include YTHDF1/2/3, YTHDC1/2 and HNRNPA2B1. The whole progression of m^6^A is summed up in Figure 1. However, additional studies need to be conducted in this area to determine other related proteins and specific regulatory mechanisms.

### 2.2 Methods for detection of m^6^A in RNAs

M^6^A methylation is abundant in RNAs, and the total level of m^6^A in cells could be detected by many methods. They are LC‐MS/MS (liquid chromatography‐tandem mass spectrometry), TLC (thin‐layer chromatography), HPLC (high‐performance liquid chromatography), m^6^A dot blot, *etc*.^7^, ^12^, ^19^, ^34^ However, there are issues to be considered, such as the requirement for high‐tech equipment, being not quantitative and too many interference factors.^7^, ^19^, ^34^ And detecting the precise site of m^6^A in RNA is hindered by the following facts. M^6^A shares nearly identical chemical properties with adenine, and it is non‐stoichiometric.^6^ In addition, M^6^A could also reverse‐transcribe to a thymine and it would not alter the coding capacity of transcripts.^6^, ^14^ In 2012, MeRIP‐seq (methylated RNA immunoprecipitation sequencing), also known as M^6^A‐seq (m^6^A‐specific methylated RNA immunoprecipitation with next‐generation sequencing), was developed for profiling the transcriptome‐wide m^6^A distribution.^6^, ^35^ In this method, mRNA was fragmented into 100‐nt‐sized oligonucleotides and immunoprecipitated with an m^6^A‐specific antibody. Libraries were prepared from immunoprecipitated m^6^A‐containing RNAs and then subjected to next‐generation sequencing. As it relies on RNA fragmentation, its resolution is around 100‐200 nt, making it hard to determine the precise locations of m^6^A in RNA and losing much stoichiometry information.^6^, ^36^ In order to achieve a higher resolution, many methods are developed, such as PA‐m^6^A‐Seq (photo‐crosslinking‐assisted m^6^A‐sequencing) and SCARLET (site‐specific cleavage and radioactive‐labelling followed by ligation‐assisted extraction and thin‐layer chromatography).^37^, ^38^ However, there still exist problems including time consumption and being unable to be subject to high‐throughput applications.^38^ Currently, the most effective method probably is MiCLIP (methylation individual nucleotide resolution crosslinking immunoprecipitation), which could detect m^6^A at precise position. In this method, m^6^A‐containing RNA is fragmented and crosslinked to anti‐m^6^A antibody under UV light, then the antibody‐RNA complexes are recovered by protein A/G‐affinity purification and RNA fragments are reverse transcribed, generating mutations or truncations in the resulting cDNA which helps to identify the precise sites of m6A residues.^39^ Researchers now are equipped with more methods to detect m^6^A; however, with so many challenges and difficulties, new methods are still in urgent need.

### 2.3 The biological functions of m^6^A

The study of m^6^A is still a nascent field of epigenetics and is currently widely recognized, with growing evidence that its reversible progress controls and determines cell growth and differentiation in regulation of several physiological processes including circadian rhythms,^40^ spermatogenesis, metabolism, embryogenesis 41 and physical developmental processes.^42^ Impaired gene regulation plays a critical role in a wide range of disorders. As the most abundant internal and reversible post‐transcriptional modification in mammalian cells, m^6^A triggers interests in evaluating the correlation between RNA regulation and human diseases. Changes in writers, erasers and readers will also have a profound influence on health. A large number of studies have demonstrated that aberrant m^6^A modification may lead to a variety of diseases, such as obesity,^43^ type 2 diabetes mellitus^44^ and infertility.^12^ Emerging evidence has indicated that m^6^A modification plays a significant role in certain cancers. However, the specific regulatory role of m^6^A in tumorigenesis and cancer progression needs to be fully elucidated. In this review, we will give an overall summary of m^6^A in the regulation of cancers.

## 3 THE DUAL ROLE OF m^6^A MODIFICATION IN HUMAN CANCERS

Accumulating evidence supports the fact that the aberrant level of m^6^A is strongly associated with a variety of cancers, such as acute myeloid leukaemia (AML), breast cancer, glioblastoma and lung cancer. The m^6^A‐associated proteins are the dominant factors in the regulation of carcinogenesis and tumour progressions. However, these proteins present a significant tumour specificity in variable tumours, which consequently contributes to the dual role (inhibition or promotion) of m^6^A modification in cancers. Upon alteration of m^6^A regulatory genes or a change in expression of proteins related to m^6^A methylation, the level of m^6^A on targeting gene mRNA would be dramatically changed and would therefore exert a profound impact on cancer development. In this section, we will systematically review the bi‐aspects of m^6^A regulating different types of cancers.

### 3.1 m^6^A modification inhibits the tumour progression

Emerging evidence has demonstrated that up‐regulation of m^6^A could inhibit tumour progressions in several types of cancers. Consistently, it was observed that m^6^A obtained low expression in cervical cancer, and the reduced m^6^A level was associated with higher FIGO stage and recurrence.^45^ Further investigation showed that down‐regulation of m^6^A level induced by interference of METTL3 and METTL14 or overexpression of FTO and ALKBH5 could promote cervical cell proliferation, and *vice versa*. Herein, we conclude the possible mechanisms about m^6^A‐associated proteins in other cancers as follows.

#### 3.1.1 METTL3

As an important “writers”, several studies have indicated the tumour suppressor role of METTL3 with up‐regulating m^6^A modification. However, the role of m^6^A is controversial in glioblastoma. Glioblastoma is an aggressive primary brain tumour in adults and has no significant improvement in survival rate so far.^46^ Previous studies strongly indicated the role of m^6^A methylation in self‐renewal and tumorigenesis of glioblastoma stem cells (GSCs).^47^ Reduction in m^6^A level induced by METTL3 silencing led to the up‐regulation of a group of oncogenes such as ADAM19, EPHA3 and KLF4, but a down‐regulation of tumour suppressors including CDKN2A, BRCA2 and TP53I11. Besides, in terms of phenotypes, knockdown of METTL3 promoted GSCs growth and self‐renewal as well as tumour progression, and *vice versa*.^47^ It indicated that METTL3 was possibly a tumour suppressor of glioblastoma. However, recently, Visvanathan et al. have discovered powerful but opposite evidence that METTL3‐mediated m^6^A modification was required for GSCs maintenance.^48^ The underlying mechanism here is controversial with what we discussed above. The results indicated that METTL3 was up‐regulated in GSCs, whereas METTL3 silencing down‐regulated the glioma reprogramming factors POU3F2, SOX2, SALL2 and OLIG2 and inhibited the growth of GSCs.^48^ Further RNA immunoprecipitation studies identified that METTL3 methylated specific sites of SOX2‐3′UTR and that the recruitment of HuR to m^6^A‐modified sites was essential for SOX2 mRNA stabilization. In addition, the characteristic of radio‐resistance in GSCs showed a positive relationship with the level of METTL3, in which SOX2 played a regulatory role.^48^

#### 3.1.2 METTL14

METTL14 performed tumour suppressor functions similar to that of METTL3 in the development of GSCs by targeting mRNA levels of ADAM19, EPHA3 and KLF4.^47^ Wang et al. reported that METTL14‐knockdown GSCs showed less demethylation changes as compared to METTL3‐knockdown GSCs.^49^ For hepatocellular carcinoma (HCC), the METTL14 expression and m^6^A level exhibited a converse tendency with the development of HCC, particularly in metastatic HCC. The clinical data also revealed that patients with decreased METTL14 showed worse recurrence‐free survival and overall survival.^50^ METTL14 deficiency enhanced the metastatic capacity of HCC cells, and conversely, overexpression of METTL14 suppressed cell migration and invasion.^50^ Further experiments demonstrated that pri‐miR‐126 was a direct target of METTL14 with m^6^A modification and m^6^A‐modified pri‐miR‐126 bound to DGCR8 to induce the expression of mature miR‐126 consequently. Induced mature miR‐126 was finally identified as a tumour suppressor.^50^ It was also discovered that METTL14 presented a markedly decreased tendency in breast cancer^50^ and miR‐126 was also recognized as a metastasis suppressor of breast cancer,^51^ which indicated that METTL14 possibly regulated breast cancer by targeting miR‐126 in a m^6^A‐dependent manner.

#### 3.1.3 FTO

FTO is well recognized for its strong association with increased body mass and obesity.^52^, ^53^ Given that obesity is a well‐established risk factor for a wide range of cancers, it is reasonable to postulate that FTO is intimately linked to cancers. In fact, a meta‐analysis revealed that various FTO SNPs were associated with different cancers such as endometrial cancer, pancreatic cancer and breast cancer dependent on or independent of BMI adjustment.^54^ In addition, aberrant expression or mutation of FTO has been shown to have an intimate link with prostate cancer,^55^ endometrial cancer^56^ and breast cancer.^57^, ^58^, ^59^, ^60^ However, the potential role of FTO as a demethylase remains a nascent yield to be explored. Just recently, the potential role of FTO in cancer initiation and progression by down‐regulating the overall m^6^A level has been investigated. In this study, Li et al. illustrated that FTO was significantly up‐regulated in certain sub‐types of AMLs such as MLL‐rearranged AML and acute promyelocytic leukaemia.^61^ Notable enrichment of FTO directly up‐regulated by the oncogenic proteins (*e.g*. MLL‐fusion proteins, PML‐RARA, FLT3‐ITD and NPM1 mutant) was observed in CD34+ bone marrow cells separated from primary MLL‐rearranged AML patients. Mechanistically, FTO exerted its oncogenic role by targeting the tumour suppressor ASB2 and RARA with its m^6^A demethylase catalytic activity. Thus, the FTO‐mediated inhibition of the ASB2/RARA axis with decreased overall m^6^A level markedly contributed to the carcinogenesis of AMLs.^61^ Recently, researchers have found the antileukaemic activity of R‐2HG, which was the production of mutant isocitrate dehydrogenase 1/2 and used to be considered as an oncometabolite, and FTO was involved in this progress. To those R‐2HG‐sensitive cells, R‐2HG could enhance the m^6^A level of MYC/CEBPA mRNA by inhibiting FTO activity. When read by YTHDF2, m^6^A‐containing mRNA was significantly degraded; thus, the R‐2HG‐FTO‐m^6^A‐MYC/CEBPA axis greatly suppresses the proliferation of leukaemia cells. And this finding also provides us a promising therapy like the combination of R‐2HG and inhibitor of MYC signalling to cure leukaemia.^62^

It is also reported that FTO is involved in the progress of glioblastoma development. MA2 is a chemical inhibitor of FTO and could increase m^6^A level in human cells.^63^ When treated with MA2, there was a dramatic inhibition of GSCs growth and self‐renewal *in vitro*, and the sphere formation rates induced by METTL3‐ or METTL14‐knockdown GSCs were also reversed.^47^

#### 3.1.4 ALKBH5

ALKBH5 is a nuclear 2‐oxoglutarate‐dependent oxygenase and is inducible by hypoxia‐inducible factor 1 (HIF‐1) in a large number of cells.^64^ Intratumoural hypoxia is commonly found in cancers and is an essential microenvironment for cancer progression.^65^, ^66^, ^67^, ^68^ A recent study has suggested that intratumoural hypoxia is a driving force for breast cancer progression.^69^ Breast cancer stem cells (BCSCs) phenotype is specified by certain core pluripotency factors including NANOG and KLF4, which could be regulated by HIF‐1.^70^, ^71^ The above findings led to an investigation of the functional significance of ALKBH5 as an RNA demethylase in cancers. Zhang et al. illustrated that exposure of a subset of breast cancer cells to hypoxia induced ALKBH5 expression in an HIF‐dependent manner, which led to reduction in m^6^A modification of NANOG mRNA and enhanced NANOG mRNA stability.^72^ In addition, ALKBH5 depletion impaired hypoxia‐induced BCSCs enrichment and tumour formation.^72^ Further study showed that ALKBH5 expression was required for breast cancer initiation and lung metastasis.^73^ Zinc finger protein 217 (also known as ZNF217) plays a complementary role with ALKBH5 in negatively regulating m^6^A methylation. ZNF217 was identified to function as the m^6^A methyltransferase inhibitor by sequestering METTL3, consequently promoting the expression and stability of NANOG mRNA and KLF4 mRNA.^73^ In addition, ZNF217 expression is also induced in an HIF‐dependent manner under hypoxic conditions. Therefore, ZNF217 depletion leads to impaired hypoxia‐induced consistent enrichment of BCSCs with ALKBH5 deficiency.^74^ In terms of glioblastoma, inactivated ALKBH5 inhibited the proliferation and tumorigenesis of GSCs and impaired GSCs self‐renewal.^75^ It is widely accepted that FOXM1 plays a pivotal role in regulating GSCs proliferation and self‐renewal.^76^, ^77^ ALKBH5 was found to demethylate FOXM1 nascent transcripts and promote FOXM1 expression, whereas long non‐coding RNA antisense of FOXM1 further promoted the interaction of FOXM1 nascent transcripts with ALKBH5. That makes ALKBH5‐FOXM1 important for glioblastoma development.

#### 3.1.5 YTHDF2

YTHDF2 is recognized as a reader protein of m^6^A methylation and mediates the m^6^A‐containing mRNA degradation. YTHDF2 was found to be up‐regulated in HCC, and miR‐145 was identified as an upstream regulatory factor to elevate m^6^A level by targeting YTHDF2 which was consistent with silencing of YTHDF2.^78^ In addition, YTHDF2 was also found to be significantly up‐regulated in prostate cancer tissues.^79^ Knockdown of YTHDF2 greatly enhanced the level of m^6^A and led to the inhibition of proliferation and migration of prostate cancer cells, whereas overexpression of miR‐493‐3p showed similar outcome.^79^ Further experiment indicated that miR‐493‐3p directly targeted the 3′UTR of YTHDF2, inhibited the YTHDF2‐induced m^6^A degradation and thus suppressed prostate cancer development. The above observations indicate that YTHDF2 is involved in cancer development by down‐regulating m^6^A level.

#### 3.1.6 SAM

S‐adenosyl‐L‐methionine (SAM) is the donor of the methylation group in m^6^A methylation reactions. Enriched abundance of SAM inhibited the growth of breast cancer,^80^ liver cancer,^81^, ^82^ colon cancer^83^ and gastric cancer cells.^84^

In summary, carcinogenesis or tumour progression in certain cancers can be significantly inhibited by up‐regulation of m^6^A modification induced by overexpression of the tumour suppressor “writer” (METTL3 and METTL14) and SAM and silencing of the oncogene “eraser” (FTO and ALKBH5) and “reader”(YTHDF2).

### 3.2 m^6^A modification promotes tumour progression

A global view depicts that mutations of m^6^A regulatory genes were identified in 2.6% of AML, 2.4% of multiple myeloma and 1.0% of acute lymphoblastic leukaemia.^85^ It was also observed that copy number variations (CNVs) appeared in 10.5% of AML patients, among which copy number loss of ALKBH5 was the most frequent. Additionally, 9 of 191 patients showed concomitant copy number gain or loss of more than one m^6^A regulatory gene. The mutations and CNVs of m^6^A regulatory genes were associated with poorer cytogenetic risk and other clinic‐pathological or molecular features in AML.^85^ In addition, impaired m^6^A regulatory genes were notably associated with the presence of TP53 mutations in AML patients and both might play a complementary role in the maintenance of AML.^85^ Collectively, it is unknown whether single alteration or multiple changes in m^6^A modification profoundly affect leukaemia. Huang et al. investigated the DNA and RNA methylation status in circulating tumour cells (CTCs) from lung cancer patients.^86^ There was a dramatic decrease in 5‐methyl‐2′‐deoxycytidine, but an increase in 5‐methylcytidine and m^6^A levels in CTCs from lung cancer patients, implying that increased m^6^A level potentially plays critical roles in tumorigenesis.

#### 3.2.1 METTL3

Regardless of its tumour suppressor role in glioblastoma, certain studies depict that METTL3 promotes cell growth, survival and invasion in several cancers. One study suggested that silencing of METTL3 in HepG2 cells was strongly associated with a noteworthy enhancement of the p53 signalling pathway.^6^ In addition, it was observed that METTL3 was drastically up‐regulated in HCC patients, with a positive correlation with the higher grade of HCC.^87^ Tumour suppressor SOCS2 was observed to be the direct downstream target of METTL3. YTHDF2 directly recognized higher m^6^A modification of SOCS2 mRNA mediated by METTL3, which subsequently induced degradation of SOCS2.^88^ Consistently, knockdown of SOCS2 drastically enhanced HCC proliferation. Collectively, up‐regulation of METTL3 suppresses the expression of SOCS2 and promotes HCC development through m^6^A modification. HBXIP is commonly identified as an oncogene and exerts a profound effect on breast cancer.^89^, ^90^ A recent study has found that there was a strongly positive association between HBXIP and METTL3.^91^ Overexpression of HBXIP could significantly elevate the expression of METTL3 in breast cancer tissues and *vice versa*. Knockdown of METTL3 additionally resulted in a reduction in HBXIP. Further investigation showed the underlying mechanism whereby HBXIP inhibited the tumour suppressor let‐7 g, which could down‐regulate METTL3. In the meanwhile, METTL3 promoted the expression of HBXIP by m^6^A modification. Thus, an HBXIP/let‐7 g/METTL3‐positive feedback loop forms, leading to the proliferation of breast cancer cells.^91^ Apart from the above, there are more encouraging findings associated with AML. It was reported that METTL3 exerted tumour promoter function in the development of AML with the methylation catalytic activity.^92^, ^93^ Knockdown of METTL3 led to a proliferation defect in AML cells, whereas overexpression of METTL3 rescued the proliferation defect, but inhibited myeloid differentiation of haematopoietic stem/progenitor cells (HSPCs).^92^, ^93^ Further investigations showed that the expression patterns of METTL3 and METTL14 were much more abundant in AML cell lines. And elevated m^6^A mediated by METTL3 played a vital role in the maintenance of the cell‐undifferentiated state in AML. Interestingly, METTL3 was identified to be recruited by CEBPZ to the transcriptional starting site of SP1, which stimulated the translation of SP1. Consequently, SP1 regulated the oncogene c‐MYC and led to the development of AML.^92^ Additionally, Vu et al. found that m^6^A mediated by METTL3 promoted the translation of c‐MYC, BCL2 and PTEN mRNAs, and the PTEN transcript may encode a negative regulator of p‐AKT which was considered to promote differentiation and inhibit self‐renewal.^93^

Although METTL3 was previously thought to be down‐regulated in GSCs, the concept has become controversial. A recent study has supported that METTL3 was up‐regulated in GSCs and that silencing of METTL3 inhibited cell growth by repressing POU3F2, SOX2, SALL2 and OLIG2.^48^ Additional investigations are supposed to be initiated to further identify the underlying mechanisms of METTL3 in GSCs.

Other studies find a new paradigm of how METTL3 affects tumour development independent of m^6^A modification.^94^ Lin et al. observed that METTL3 enhanced the translation of certain oncogenes such as EGFR, TAZ, MAPKAPK2 and DNMT3A, which bears one or more m^6^A peaks near the stop codon.^94^ However, surprisingly, METTL3 promoted translation independent of its methyltransferase activity or m^6^A readers, because the catalytic domain of METTL3 showed no effect in promoting translation of the above oncogenes. In addition, knockdown of YTHDF1/YTHDF2 did not influence METTL3‐mediated translation.^94^ These findings provide us with insight into the mechanisms of m^6^A modification‐related proteins.

#### 3.2.2 METTL14

As mentioned above, METTL14 is abundant in AML cells. Weng et al. further indicated that METTL14 was highly expressed in normal HSPCs and AMLs, which was required for leukaemia stem cell self‐renewal and maintenance.^95^ Mechanically, METTL14 positively regulated the mRNA stability and translation of oncogene MYB and MYC, which could be negatively regulated by SPI1. However, the regulation of METTL14 on MYB and MYC is not conducted by YTHDF protein as YTHDF gene showed no consistent pattern during the process of regulation.^95^ Collectively, SPI1‐METTL14‐MYB/MYC axis plays a vital role in AML development. When treated with differentiation‐inducing agents such as ATRA, the level of METTL14 and m^6^A decreased, which proposed a novel treatment mechanism of ATRA.

### 3.3 Potential association of m^6^A with other cancers

Some m^6^A‐associated proteins have been found to be involved in tumour progressions before the observation and mechanism studies of m^6^A modification phenomenon. For example, it is known that FTO plays a role in body mass and obesity.^52^, ^53^ Besides, several studies have revealed the links between various FTO SNPs and endometrial cancer, pancreatic cancer and breast cancer.^54^ However, FTO was only found to promote AML and glioblastoma progression with its “eraser” role. Other FTO‐induced cancers have not been investigated so far, and additional studies need to be performed to explore the eraser role of FTO in regulating these cancers. The situation is the same for WTAP, which has been reported to have a close relationship with cancers. WTAP was overexpressed in cholangiocarcinoma, especially in metastatic cholangiocarcinoma cells inside lymph nodes or vessels. Overexpression or knockdown of WTAP significantly increased or decreased the migration and invasion of cholangiocarcinoma cells.^96^ And it was discovered that WTAP overexpression greatly induced metastasis‐associated genes such as MMP7 and MMP28 that degrade extracellular matrix components.^96^ In addition, Xi et al. confirmed that WTAP was expressed at a significantly high level in gliomas and was closely correlated with the prognosis of patients with glioblastoma.^97^ Interestingly, WTAP was also described as a novel oncogenic protein in AML.^98^ However, the m^6^A‐associated mechanisms of WTAP in regulating the above cancers have not been elucidated. To conclude, the above evidence strongly indicates that several m^6^A‐associated proteins such as FTO and WTAP are possibly involved in the progression of certain cancers, which introduces a new functional mechanism for these proteins.

## 4 CONCLUSIONS

**Figure 2:**
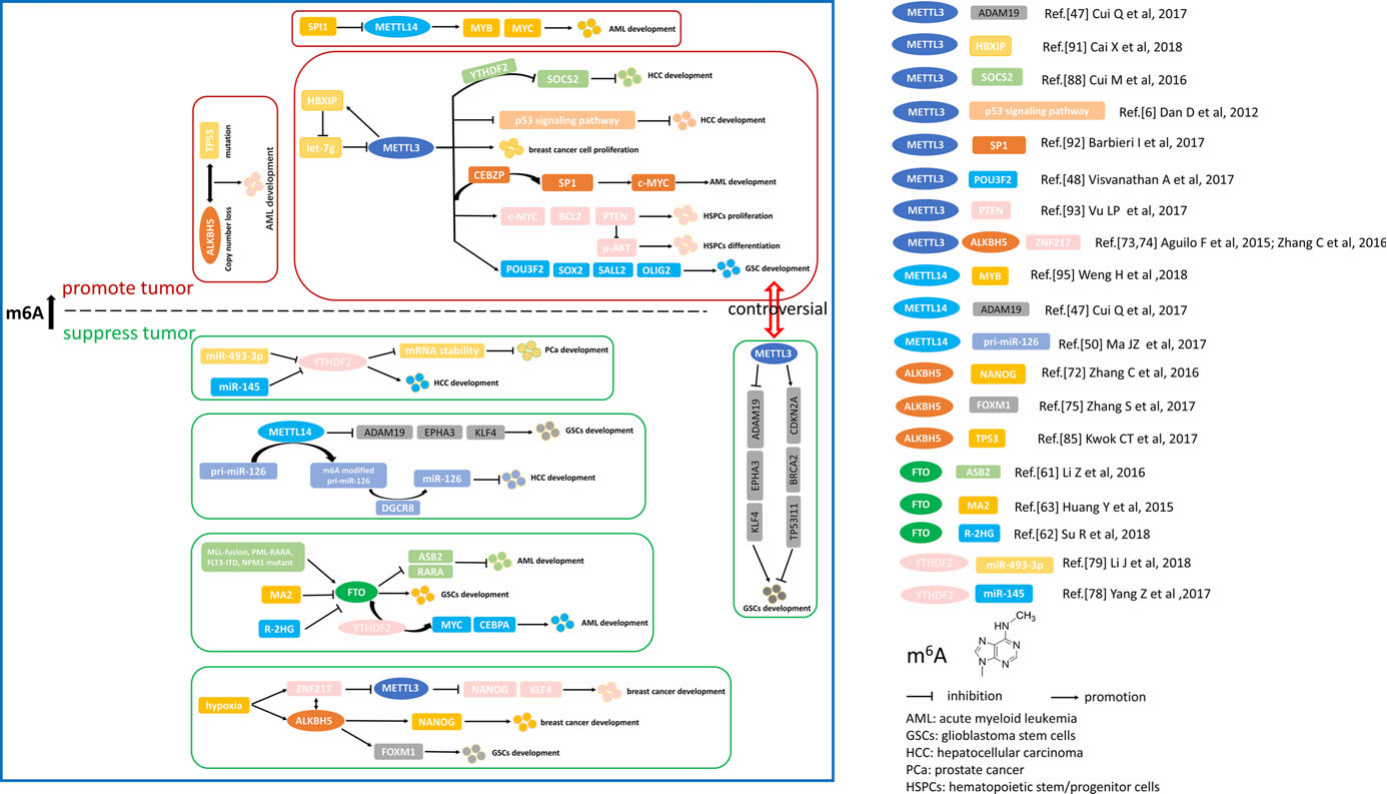
Dual role of m^6^A modification in human cancers. Aberrant expression of m^6^A modification induced by down‐regulation or up‐regulation of methyltransferases, demethylases or readers promotes or suppresses tumour development. See references for more details.

To summarize, aberrant level of m^6^A intimately links to human cancers, and mechanisms of the network of m^6^A modification and cancers are gradually being discovered, but still require further investigations. Currently, we find the dual role of m^6^A modification in the regulation of cancers which are shown in Table 1 and Figure 2. On the one hand, increased m^6^A level inhibits the carcinogenesis, and on the other hand, increased m^6^A level promotes the tumour progressions. Because of the reversible process or because m^6^A is mediated by writers, erasers and readers, the dysregulations of these proteins may trigger or be involved in the tumour progressions. In terms of mechanisms, m^6^A modification, as a critical post‐transcriptional regulation, is demonstrated to dynamically regulate RNA biology and may be a vital tumour promoter or suppressor involved in tumour progressions. The expression pattern of the same m^6^A‐associated protein and its target mRNAs may differ in multiple cancers, which represents the significant tumour specificity. Previously determined m^6^A‐associated proteins involved in cancers may need additional investigations to explore the new regulatory mechanisms according to the m^6^A way. Thus, the function of m^6^A may be broader than the existing paradigm and may provide profound insights into tumorigenesis and cancer development. However, details are still lacking and a better understanding of the basic mechanism is required. In addition, corresponding proteins that dynamically regulate m^6^A methylation require further exploration.

**Table 1:** The dual role of m6A modification in human cancers

Molecule	Cancer	Role in cancer	Biological function	Mechanism
**m^6^A modification inhibits the tumour progression**				
METTL3	GBM (glioblastoma)	Suppressor gene	Suppresses GSC (glioblastoma stem cells) growth and self‐renewal	Down‐regulates ADAM19, EPHA3 and KLF4; Up‐regulates CDKN2A, BRCA2 and TP53I11
METTL14	GBM	Suppressor gene	Suppresses GSC growth and self‐renewal	Down‐regulates ADAM19, EPHA3 and KLF4
METTL14	HCC	Suppressor gene	Suppresses HCC metastasis	Promotes pri‐MIR‐126 processing
FTO	AML	Oncogene	Promotes AML carcinogenesis	Inhibits ASB2/RARA axis
FTO	AML	Oncogene	Promotes AML carcinogenesis	Enhances MYC and CEBPA mRNA stability
ALKBH5	Breast cancer	Oncogene	Promotes breast cancer initiation	Enhances NANOG and KLF4 mRNA stability
ALKBH5	GBM	Oncogene	Promotes GBM proliferation and self‐renewal	Promotes FOXM1 expression
YTHDF2	Prostate cancer	Oncogene	Promotes prostate cancer growth and migration	Promotes m^6^A‐containing mRNA degradation
**m^6^A modification promotes the tumour progression**				
METTL3	AML	Oncogene	Promotes proliferation; Inhibits differentiation	Promotes MYC, BCL2 and PTEN translation
METTL3	Breast cancer	Oncogene	Promotes breast cancer cells proliferation	Promotes HBXIP translation
METTL3	HCC	Oncogene	Promotes HCC growth	Promotes SOCS2 degradation
METTL3	GBM	Oncogene	Promotes GSCs growth and self‐renewal	Up‐regulates POU3F2, SOX2, SALL2 and OLIG2
METTL14	AML	Oncogene	Promotes AML cells self‐renewal and maintenance	Up‐regulates MYB and MYC

As an RNA modification, m^6^A opens avenues for correlating epigenetics with diseases, especially cancer, which proposes a new mechanism in cancer regulations. In terms of the translation into clinical medicine, it will be of great importance to investigate whether competitive antagonists of those proteins can act as potential anticancer agents.

## CONFLICT OF INTERESTS

The authors confirm that there are no conflicts of interest.

## AUTHORS’ CONTRIBUTIONS

LH, JL and XW equally contributed to the collection of data, preparation of figures and drafting individual sections of the manuscript; YY, HX and HY contributed to the collection of data and writing individual sections of the manuscript; XZ and LX revised and expanded the manuscript. All authors read and approved the final manuscript.
